# Supplementary material for: Bond Strength of Universal Adhesives to Dentin: A Systematic Review and Meta-Analysis
Source: Polymers (Basel). 2021 Mar 7;13(5):814. doi: 10.3390/polym13050814 (PMC7961712; doi:10.3390/polym13050814)
Supplement: Supplementary file 1 [file polymers-13-00814-s001.pdf]

Table S1. Search strategy used in ISI Web of Science.

	Search strategy
# **1**	ALL= (Universal adhesives OR Universal adhesive OR Universal simplified adhesive systems OR Universal Dental Adhesives OR Multipurpose adhesives OR multi-purpose adhesives OR multimode adhesives OR multi-mode adhesives OR universal bonding agent OR multi purpose adhesives OR multi mode adhesives)
# **2**	ALL= (Bond OR Bonding OR dental bonding OR Bonding efficacy OR bond strength OR Bonding performance OR bonding effectiveness OR Bond performance OR adhesive properties OR microtensile strength OR Micro-tensile strength OR bonding properties OR Microtensile bond strength OR shear bond strength OR microshear bond strength OR Performance)
# **3**	ALL= (dentin or dentine)
# **4**	#1 and #2 and #3

Table S2. Search strategy used in SCOPUS.

	Search strategy
# **1**	ALL ("Universal adhesives" OR "Universal adhesive" OR "Universal simplified adhesive systems" OR "Universal Dental Adhesives" OR "Multipurpose adhesives" OR "multi-purpose adhesives" OR "multimode adhesives" OR "multi-mode adhesives" OR "universal bonding agent" OR "multi purpose adhesives" OR "multi mode adhesives") AND ALL("Bond" OR "Bonding" OR "dental bonding" OR "Bonding efficacy" OR "bond strength" OR "Bonding performance" OR "bonding effectiveness" OR "Bond performance" OR "adhesive properties" OR "microtensile strength" OR "Micro-tensile strength" OR "bonding properties" OR "Microtensile bond strength" OR "shear bond strength" OR "microshear bond strength" OR "Performance") AND ALL("dentin" or "dentine")

Table S3. Search strategy used in EMBASE.

	**Search strategy**
**# 1**	'universal adhesives' OR 'universal adhesive' OR 'universal simplified adhesive systems' OR 'universal dental adhesives' OR 'multipurpose adhesives' OR 'multi-purpose adhesives' OR 'multimode adhesives' OR 'multi-mode adhesives' OR 'universal bonding agent' OR 'multi purpose adhesives' OR 'multi mode adhesives'
**#2**	'bond' OR 'bonding' OR 'dental bonding' OR 'bonding efficacy' OR 'bond strength' OR 'bonding performance' OR 'bonding effectiveness' OR 'bond performance' OR 'adhesive properties' OR 'microtensile strength' OR 'micro-tensile strength' OR 'bonding properties' OR 'microtensile bond strength' OR 'shear bond strength' OR 'microshear bond strength' OR 'performance'
**#3**	'dentin' OR 'dentine'
**#4**	#1 and #2 and #3

Table S4. Excluded studies

Study	Reason for exclusion
Al Deeb, 2020	Universal adhesive was not evaluated
Anja, 2015	Universal adhesive was not evaluated.
Alaghehmand, 2016	Universal adhesive was not evaluated
Cardenas, 2018	Universal adhesive was not evaluated
Chiang, 2013	Universal adhesive was not evaluated
De Alencar, 2014	Universal adhesive was not evaluated
Deng, 2016	Universal adhesive was not evaluated
Devarajan, 2020	Universal adhesive was not evaluated
Feiz, 2017	Universal adhesive was not evaluated
Gu, 2019	Universal adhesive was not evaluated (experimental adhesive was used)
Shahabi, 2013	Universal adhesive was not evaluated
Yan, 2018	Universal adhesive was not evaluated
De Siqueira, 2020	Universal adhesive was not used using any additional technique.
Sinjari, 2020	Universal adhesive was not used using any additional technique.
Lee, 2013	Universal adhesive was not used using any additional technique.
Khatib, 2020	Adhesive used could not be identified.
Martini, 2017	Sclerotic dentin was used as a bonding substrate
Zumstein, 2018	Eroded dentin was used as a bonding substrate
Santos, 2017	Full-text could not be retrieved
Tsujimoto, 2017	Full-text could not be retrieved
Ouchi, 2020	Bovine dentin was used as a bonding substrate
Poggio, 2017	Bovine dentin was used as a bonding substrate
Tamura, 2017	Bovine dentin was used as a bonding substrate
Santos, 2016	Bovine dentin was used as a bonding substrate
Şişmanoğlu, 2019	Bovine dentin was used as a bonding substrate
Souza, 2018	Bovine dentin was used as a bonding substrate
Ayar, 2016	Bovine dentin was used as a bonding substrate
Moritake, 2018	Bovine dentin was used as a bonding substrate
Saito, 2020	Bovine dentin was used as a bonding substrate
Da Rosa, 2018	Bovine dentin was used as a bonding substrate
Tsujimoto, 2016	Bovine dentin was used as a bonding substrate
